# Quantitative free-breathing 3T T_2_-mapping of the heart designed for longitudinal studies

**DOI:** 10.1186/1532-429X-14-S1-O51

**Published:** 2012-02-01

**Authors:** Ruud B Van Heeswijk, Hélène Feliciano, Gabriele Bonanno, Simone Coppo, Nathalie Lauriers, Didier Locca, Juerg Schwitter, Matthias Stuber

**Affiliations:** 1 Radiology, University Hospital Lausanne (CHUV), Lausanne, Switzerland; 2 Center for BioMedical Imaging (CIBM), Lausanne, Switzerland; 3 Center for Cardiac Magnetic Resonance and Cardiology Service, University Hospital Lausanne (CHUV), Lausanne, Switzerland

## Background

Recently, T_2_-weighted MRI for the characterization of edema after myocardial infarction has attracted considerable attention (Friedrich, NatRevCardiol2010). Furthermore, the recently proposed combination of bSSFP imaging and T_2_Prep for T_2_-mapping at 1.5T has enabled a rapid quantitative cardiac T_2_ estimation (Huang et al., MRM2007). However, the accuracy of this method may still be limited due to the complex T_2_/T_1_ signal weighting. Especially for longitudinal studies designed for monitoring and/or guiding therapy, accurate and reproducible T_2_ measurements will be critical. A novel quantitative 3T T_2_-mapping protocol was therefore developed and tested in both healthy volunteers and patients.

## Methods

An adiabatic T_2_prep with 3 incremental TE values, affine coregistration, a navigator and 2D radial gradient echo imaging were combined for free-breathing T_2_-mapping at 3T with a spatial resolution of 1.25mm. Bloch equation simulations of this sequence were used to optimize scan parameters and to determine an empirical equation that compensates for T_1_ relaxation and which returns the “true” T_2_. The T_2_-mapping sequence and empirical equation were then validated in a series of 15 phantoms in which the true T_2_ was determined with a 9-TE spin-echo sequence. Next, the myocardial short axis T_2_ of 8 healthy volunteers was mapped in two different scan sessions while a reference phantom (T_2_=43.1±0.7ms) was placed next to the thorax. The average myocardial T_2_ for both sessions was computed with and without correction with the “true” reference phantom T_2_. Finally, this validated protocol was used in 5 patients in the subacute phase after revascularization of acute ST-elevation myocardial infarctions and compared to T_2_-weighted TSE imaging.

## Results

**Figure 1:**
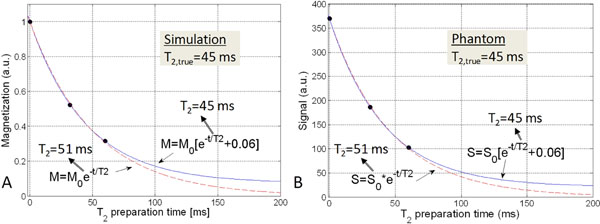
Single pixel T_2_-mapping in a simulation and phantom scan. A) Simulated magnetization (black dots) for myocardium with input T_2_=45ms at the T_2_prep times (60, 30 and 0ms) and fitted curves with the standard (dashed line) and new, empirical (whole line) equation. The new equation leads to more accurate T_2_ computations. B) Similar results are obtained in a pixel in a T_2_map of a phantom where the T_2_ was determined to be 45ms with a 9-TE spin echo scan.

**Figure 2:**
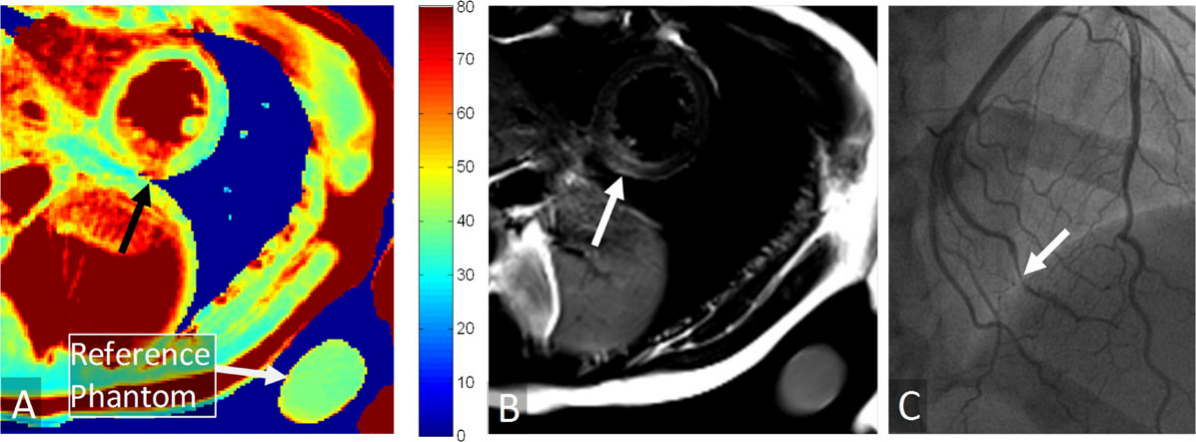
Short-axis T_2_map together with conventional T_2_-weighted turbo spin-echo and X-ray coronary angiogram in a patient with a myocardial infarct. A) A clearly demarcated zone with elevated T_2_ can be seen in the region of the black arrow, which might indicate myocardial edema. The non-infarcted tissue has a homogenous T_2_, while the reference phantom adjacent to the thorax appears homogeneous with T_2_ values similar to those in healthy tissue. B) The conventional T_2_-weighted TSE image confirms the elevated T_2_ in the region of the infarct (arrow). C) Consistent with these findings, the x-ray coronary angiogram shows a severe stenosis in an obtuse marginal artery (arrow).

As a result of both the simulations and phantom scans, optimized sequence parameters included: TE_T2prep_=60/30/0ms, T_RR_=3 heartbeats, TR/TE=5.3/2.4ms. The empirical equation to determine T_2_ was S=S0[exp(-TE_T2prep_/T_2_)+0.06], where S and S0 are the measured and steady-state signal (Fig. 1a). Scans of the phantoms with known T_2_ confirmed a 12±2%(p<0.001) improvement in T_2_ estimation with the empirical equation as compared to the standard T_2_ decay measurements (Fig. 1b). The myocardial T_2_ in the volunteers was homogeneous (42±5ms over all volunteers) and on average showed a 5±2% difference between the two scan sessions. When compensated with the T_2_ from the reference phantom, this difference decreased to 2±1% (p=0.02). In all patients, T_2_maps could successfully be obtained and a clear demarcation of zones with elevated T_2_ values was consistent with the findings on T_2_-weighted MRI and X-ray coronary angiography as shown in the example in Fig. 2.

## Conclusions

The methodology presented in this study enables robust and accurate cardiac T_2_-mapping at 3T, while the addition of a reference phantom improves reproducibility. Therefore, it may be well-suited for longitudinal studies in patients with ischemic heart disease.

## Funding

N/A.

